# Evaluation of Commercial Meat Products of Red Chicken Reared under LED Lights

**DOI:** 10.3390/foods11030370

**Published:** 2022-01-27

**Authors:** Martina Colapietro, Andrea Ianni, Francesca Bennato, Giuseppe Martino

**Affiliations:** Faculty of Bioscience and Technology for Food, Agriculture and Environment, University of Teramo, 64100 Teramo, Italy; mcolapietro@unite.it (M.C.); aianni@unite.it (A.I.); fbennato@unite.it (F.B.)

**Keywords:** light-emitting diodes, breast meat, fatty acid, volatile profile

## Abstract

**Simple Summary:**

The aim of the study was to investigate the role of three different light-color temperatures of Light-Emitting Diodes (LEDs) (Neutral, Cool and Warm) on some attributes of breast meat. Various changes were observed in the physical and chemical characteristics of breast meat samples and the results obtained in fatty and volatile profiles indicate that the LEDs change the characteristics of meat.

**Abstract:**

The objective of our study was to investigate the role of three different light-color temperatures of Light-Emitting Diodes (LEDs) [Neutral (K=3300−3700); Warm (K=3000−2500) and Cool (K=5500−6000)] on the qualitative attributes of breast meat obtained from male AZ Extra Heavy Red chickens. The comparison was made with meat deriving from chickens reared in the presence of classic neon lighting (Control). The meat was analyzed for the determination of both physical and chemical properties (cooking loss, moisture, total lipids and fatty acid composition). Furthermore, meat samples subjected to cooking were also analyzed for the identification of volatile compounds produced during the process; such evaluation was performed both immediately after cooking (T0) and after 7 days (T7) of cooked-meat storage at 4 °C. Cooking-loss values were higher for samples from chickens raised with Neutral LED (*p* < 0.05) compared to the other groups. For the fatty acid profiles of the meat, higher values were found for monounsaturated fatty acids (MUFAs) such as C18:1, C9 and C16:1 in Cool LED compared to the Control. Regarding the volatile profile of cooked meat, compounds belonging to the families of aldehydes, alcohols, ketones, and aromatic compounds were identified. Compounds belonging to the aldehyde family, such as hexanal, increased in Cool LED samples at T0 in comparison to the Control. On the other hand, the amounts of 1-Pentanol, 1-Octanol and 2-Octen-1-ol, which belong to the alcohol family, increased at T7 in Cool LED samples compared to the Warm LED. In conclusion, LED lighting showed to be effective in inducing significant variations on chicken breast meat ready to be introduced to the market, in particular regarding fatty acid profiles and the accumulation of volatile compounds. However more in-depth evaluation is needed for the identification of modifications regarding the sensorial sphere, which could have an impact on the consumer acceptability of the product.

## 1. Introduction

In recent years, consumption of low-fat, low-calorie, high-protein chicken meat has increased significantly [1]. The quality of poultry meat depends on the production performance of the poultry. In addition to animal genetics, the husbandry system heavily affects the poultry production performance and derived meat quality. The use of new technologies allows the farmer to increase production while reducing costs and the negative impact on the environment. Light (intensity, photoperiod, and wavelength) is essential as it has a direct influence on the behavior, physiology, immunity and consequently on the performance of the poultry [2]; all this depends on the ability of the chickens to perceive a wide spectrum of colors. Light visible to birds has a wavelength of 380 nm to 740 nm, which is between invisible infrared rays (longer wavelengths) and invisible ultraviolet light rays (shorter wavelengths) [3]. The light source for poultry farms is artificial light. Recently, the use of LEDs has gained increasing interest due to its good results in terms of energy consumption and animal performance and welfare. The use of LEDs is becoming increasingly popular on chicken farms. Energy saving, longer life and color variety are the main features of LED lights [4].

Many studies have been conducted on the effects of LED lights on many chicken parameters such as growth performance, welfare, meat quality and muscle tissue. The term “meat quality” includes all the physical, chemical, morphological, biochemical, microbial, sensory, technological, hygienic, nutritional and culinary characteristics [5].

Various studies have been carried out correlating the LED light with the quality of the meat. Karakaya et al.(2009), found that the chest and thigh muscles obtained from poultry reared with a mixed green–blue lighting group had a softer structure given the higher penetrometric values (PV) [6]. Furthermore, from the results obtained from the studies conducted by Kim et al (2013) they observed that the birds raised under the red LED had a high fat content while the white LED had the lowest percentage of fat in the breast meat, and the white light improves the content of essential and non-essential amino acids of breast meat [7]. Due to its high nutrient content and relatively low calories, poultry meat appears to increasingly meet the needs of consumers who are becoming more aware of the nutritional value of the foods they eat [8]. In the literature, there is no inherent information about the effect of LEDs on red chicken meat quality. In fact, the aim of this study was to investigate the effect of LED lights with three different color temperatures, Neutral (K=3300−3700), Cool (K=5500−6000) and Warm (K=3000−2500), on the quality red chicken meat.

## 2. Materials and Methods

### 2.1. Experimental Design and Samples Collection

The study was carried out on chicken meat ready to be introduced on the market. Meat was obtained from a farm located in the Abruzzo region (Italy) that adopted the LED lighting in some of the sheds dedicated to the breeding of AZ Extra Heavy Red chicken. In particular, the company introduced three different shades of LED lighting (Neutral (K=3300−3700); Warm (K=3000−2500) and Cool (K=5500−6000)) and this led to the need to understand if this aspect could have a direct effect on the quality parameters of chicken meat. For this purpose, the company supplied a total of 120 chicken breasts divided into 4 groups of 30 samples each: 30 samples (chicken breasts) from animals reared with classic neon lighting (Control group) and 30 for each of the 3 different types of LED lighting (experimental groups: Neutral, Cool and Warm). The chicken breasts supplied by the company were, in effect, cuts of meat ready for their introduction on the market and, for that reason, were supplied individually packaged in polystyrene trays covered with a plastic film. The study therefore aimed to make a comparison between the Control group and the experimental groups. Specifically, the meat was analyzed for the determination of both physical and chemical properties (cooking loss, moisture, total lipids and fatty acid composition) and samples not immediately used were packed under vacuum and stored at −20 °C for subsequent evaluation. Meat samples were also subjected to cooking and analyzed for the identification of the volatile compound produced during the process; such evaluation was performed both immediately after cooking (T0) and after 7 days (T7) of cooked-meat storage at 4 °C.

### 2.2. Cooking Loss and Chemical Composition of Breast Meat

Cooking loss was used for characterizing the ability of meat to retain water during cooking. Meat samples were weighed and cooked in a water bath until the core temperature was 70 °C. The temperature was monitored through the probe. The samples were then cooled to room temperature for two hours and weighed. Cooking loss was expressed as a percentage of the weight of the raw initial sample. Meat moisture and fat content were evaluated according to the methods of AOAC (2000) [9].

### 2.3. Fatty Acid Profile of Breast Meat

Five *g* of raw meat, stored at 20 °C, was homogenized, in accordance with Folch’s method (1957) [10], using Ultra-turrax-T25 with 45 mL of Folch’s solution (chloroform: methanol, 2:1). The homogenized samples were then transferred to a flat-bottomed flask and shaken for 7 hours at room temperature in the dark. All samples were transferred to a separatory funnel with the addition of 15 mL of 1% NaCl and left overnight. Using a rotary evaporator set at 40 °C, the chloroform phase was brought to dryness to obtain the total fat of the sample. 60 mg of each fat sample was mixed with 1 mL of hexane and 500 μL of sodium methoxide to obtain fatty acid methyl ester (FAME). FAME Detection was performed using a gas chromatograph (Focus GC; Thermo Scientific, Waltham, MA, USA) equipped with a capillary column (Restek Rt-2560 Column fused silica 100 m × 0.25 mm high polar phase; Restek Corporation, Bellefonte, PA, USA) and a flame ionization detector (FID). Hydrogen was used as the carrier gas. The thermal program was performed as previously described by Bennato et al. [11]. Quantification of peak areas was performed using ChromeCard software (Thermo Fisher Scientific, Milan, Italy) and the relative value of each FA was expressed as a percentage of the total FAME. Once the value of each FA was obtained, it was used to calculate the sum of saturated fatty acids (SFA), monounsaturated fatty acids (MUFA), and polyunsaturated fatty acids (PUFA).

### 2.4. Determination of Volatile Components of Cooked Breast Meat

Five grams of minced meat were previously weighed and mixed with 10 mL of a saturated aqueous NaCl solution (360 g/L) and 10 μL of internal standard solution (3-methyl-2-heptanone; 10 μg/L in ethanol) was added. A solid-phase microextraction fiber (divinylbenzene-carboxylic polydimethylsiloxane; length: 1 cm; film thickness: 50/30 m; Sigma-Aldrich, Milan, Italy) was used to perform headspace extraction of volatile compounds (VOC) with an exposure time of 60 minutes at 60 °C. The extracted VOCs were then thermally desorbed in a Clarus 580 gas chromatograph (Perkin Elmer, Waltham, MA, USA) equipped with an Elite 5MS column (inner diameter length: 30 × 0.25 mm; film thickness: 0.25 μm; Perkin Elmer, Waltham, MA, USA) and coupled to a mass spectrometer (SQ8S; Perkin Elmer, Waltham, MA, USA). Heating program and identification of VOCs were made as described above [12].

### 2.5. Statistical Analysis

All the assessments described were performed on 15 samples of meat (randomly selected) per group, with analyses performed in triplicate on the single sample. Results were expressed as means with corresponding standard deviations (SD). Data were tested for normal distribution and analyzed using the Sigma-Plot12.0 software (Systat software Inc., San Jose, CA, USA). The Anova model was used for statistical analysis, using the effect of LED light as a factor of variation. Significant differences among treatments were performed through post-hoc Tukey test; *p*-values (p≤ 0.05) were considered to be statistically significant.

## 3. Results

### 3.1. Physical and Chemical Characterization of Chicken Breast Meat

As reported in Table 1, cooking-loss values of Neutral (p<0.05) LED samples were higher than Warm LEDs, Cool LEDs and the Control samples. Dry matter and total lipid content were not different among the groups.

### 3.2. Fatty Acid Profile

Table 2 shows the fatty acid profile of the breast meat samples. The use of LEDs did not lead to significant changes in the total SFA content. In fact, it was observed that the concentration of stearic acid (C18:0) and behenic acid (C22:0) were lower or similar to the Control. Significant variations were observed for (C18:0) between the Control and Cool LED and for (C22:0) between the Neutral and Cool LED. In contrast, in Cool LED samples, an increase in MUFA (p<0.05) was observed compared to the Control. A significant increase (p<0.05) was observed in palmitoleic acid (C16:1) and oleic acid (C18:1, cis9) in the Cool LED meat samples compared to the Control. Finally, the use of Warm LEDs decreased the content of PUFA (p<0.05) compared to all the other groups, but a significant increase was observed in linolenic acid (C18:3) in Cool LEDs compared to the Warm LEDs and a significant decrease of arachidonic acid (C20:4) was observed in the Cool LEDs compared to the Control and Neutral LEDs.

### 3.3. Volatile Profile of Cooked Meat

Twenty-one compounds belonging to the family of aldehydes, alcohols, ketones, aromatic compounds and esters were found in the cooked chicken meat (T0 and T7) as shown in Table 3. At T0, the concentration of hexanal (p<0.05) increased significantly in Cool LEDs compared to the Control, but the concentrations of octanal decreased in Neutral LEDs compared to the Control (p<0.05). At T7, pentanal, 2-heptanal, heptanal (p<0.05) increased in the Cool LED samples compared to the Control, decanal (p<0.01) increased in the Cool LEDs compared to Neutral LEDs; nonanal (p<0.05) decreased in the Neutral LEDs compared to the Control. The situation was different for the family of alcohols; here, significant decreases of 1-Octen-3-ol (p<0.05) in Warm LEDs with respect to the Control, 1-Octyn-3-ol and 1-Pentanol (p<0.01) in Warm LED respect to the Neutral LEDs were observed in T0 samples; a completely opposite situation was observed at T7, where there was a remarkable increase in 1-Pentanol, 1-Octanol and 2-Octen-1-ol (p<0.05) in Cool LED treatments compared to the Warm LED ones. Regarding ketones, there was a significant increase in the Neutral and Cool LED groups compared to the Control at both T0 and T7; specifically, 2-methyl-3-octanone (p<0.05) at T0 and 2-heptanone (p<0.01) at T7.

## 4. Discussion

In our study, particular attention was paid to the characterization of the chemical composition, fatty acids and volatiles profiles, important properties of meat that influence the quality of chicken meat, and consequently the choice of the product by the consumer. In fact, the purpose of the study concerned an investigation on products intended for trade and not on the zootechnical side. In Neutral LED samples the results of cooking loss were higher. The ability to retain water in meat is influenced by a series of factors such as pH, length of the sarcomere, ionic strength and osmotic pressure, which act by altering the cellular and extracellular components and causing a reduction in the space between the myofibrils with a consequent decrease in capacity to retain water [13]. The molecular mechanism underlying the increase in cooking-loss values has not been investigated in our samples; however, as observed by Cao et al. [14], the cross-sectional area and density of myofibers in broiler chickens could change depending on the light in which they were reared. Indeed, the area of myofibers of broilers exposed to blue light was larger, suggesting that light may affect the growth of myofibers in the skeletal muscle of broilers and consequently the ability to retain water. In other species, as reported by a study (Zuo et al.) [15], on the longissimus thoracis of the yak, high levels of cooking loss were found to be correlated with high levels of expression of some genes such as desmin, troponin-T and lactate dehydrogenase. In fact, in yaks with a high cooking-loss score, it increased the level of LDHA, an enzyme that catalyzes the conversion of lactate to pyruvate. This enzyme is found in muscle and its activity tends to increase after slaughter, correlating with a decrease in pH due to an accumulation of lactate in the tissues. This reduces the ability of the meat to retain water. Preoteomic and bioinformatic studies [15] have shown that this gene tends to form networks with structural proteins such as desmin, because they are able to promote the shrinkage of myofibrils and displace water from them [16].

Among the fatty acid composition results of our research, Warm light samples had a reduction in total PUFA compared to the other LED light groups and the Control. In studies by Pinchasov on broilers, it was demonstrated that an increase in PUFA can affect the suppression of the synthesis of MUFA by inhibiting the action of the 9-desaturase enzyme, which is the main enzyme responsible for the conversion of SFA to MUFA [17]. This claim is consistent with our results, because the total amount of MUFA in Neutral and Cool LEDs was lower than in Warm LEDs. These results might suppose that LED light changed the expression of some desaturase. Cool LED samples had a significant increase of C18:1, cis9. In chickens, SCD (Stearoyl-CoA-dasaturase) catalyzes the desaturation of palmitic and stearic acids in palmitoleic acid (C16:1), and oleic acid (C18:1), respectively, via the initial desaturation of saturated fatty acids to monounsaturated fatty acids [18]. The data obtained show how the amount of the substrates of the enzyme, stearic acid, is lower in the Cool light than in the other LED light and in the Control, and consequently the high amount of oleic acid in the Cool group can be confirmed. This particular situation was observed for the results obtained for alfa-linolenic acid (C18:3) and arachidonic acid (C20:4) in Cool LEDs. These fatty acids (C18:3, C20:4) can be synthesized from C18:1 through the ELOV5 isoform elongation enzyme [19]. In our study, the amount of C18:3 is higher for the Cool LED group, but it was not for C20:4 because that was lower than other groups. In a study conducted by Nuemberg et al. [20] on pigs, an increase in n-3 PUFA, especially alfa-linoleic acid in the muscle, may cause a substantial decrease in arachidonic acid because of the action of delta-6/5-desturase enzymes in the elongation and desaturation metabolism.

The production of flavors and aromas in cooked chicken meat comes from thermal lipid degradation processes along with the Maillard reaction. Obviously, the taste of cooked meat is influenced by several pre- and post-slaughter factors, including breed, diet, post-mortem ageing, and cooking method [21]. In this study 21 VOC were detected in cooked-meat samples obtained from chickens reared under all experimental conditions. Chicken meat contains a higher proportion of unsaturated fatty acids than red meat. This characteristic makes the meat more susceptible to quality deterioration due to the oxidation of lipids, which leads to the formation of products such as aldehydes, ketones, alcohols, aliphatic hydrocarbons, acids, and esters, which are responsible for the development of aromatic substances in meat [22,23]. After cooking and after 7 d of storage of the cooked product, the most represented compounds belong to the aldehyde family; this result is related to the degradation of PUFAs during cooking, which give rise to the VOCs through the activity of lipolysis [24].

Our results show that at T0, the amount of hexanal was high in all LED light samples. On the other hand, at time T7, an increase in the amount of pentanal, heptanal and decenal was observed in all three LED light groups. Pentanal and hexanal prompted beta-oxidation of fatty acid, mainly alfa-linolenic acid [25], and are also indicators of meat flavor deterioration [26]. Moreover, as shown by Guerrero-Lagarreta, some aldehydes (hexanal, pentanal, heptanal, octanal, and nonanal) are responsible for unpleasant odors. This suggests that at time T7, chicken meat was more susceptible to the production of unpleasant odors than T0. A significant decrease in all experimental groups was represented by 1-octen-3-ol belonging to the alcohol family that provided fishy, fatty, mushroom, and grassy odors. It showed a lower amount in both T0 and T7 in all LED light samples than in the Control. This compound derived from an enzymatic reaction similar to lypoxygenases and hydroperoxidase lyases. Del Pulgar et al. [24] argue that a lower number of these compounds in cooked meat could depend on a lower amount of PUFA in meat, which is consistent with the results obtained, since the amount of PUFA in the experimental groups is lower than in the Control. Since at T7 for 1-pentanol, 1-octanol, 2-octen-1-ol, a higher concentration was shown for the Neutral and Cool group compared to the Control, but not for Warm LED, in agreement with the study carried out by Bennato et al. [11], Warm LED is the best condition for limiting oxidative processing, the main process in which these compounds are formed.

This study highlighted the differences between the amount of two-compounds (2-Hepatanone, 2-methyl-octanone) belonging to ketones family. Under exposure to Warm light, it is noted that at T7, 2-Heptanone tends to decrease compared to the other LED lights and the Control. Ketones are considered the responsibility of the onset of off-flavors and off-odors. This can originate from Maillard compounds [27] and from lipolytic activity and β-oxidation [28]. The very likely absence of oxidative processes can easily explain the lack of variation in ketone compounds in Warm light samples compared to the Control and other LED group.

## 5. Conclusions

The study showed the ability of LED lights to modify the fatty acids and volatile profile of the breast meat of red chickens. In the Cool LED group a significant increase of MUFA, particularly of oleic acid, was observed. The MUFA appears to be very good for human health. On the other hand, based on the cooked-meat volatile profile, the high amount of hexanal at T0 and the increase of aldehyde compounds, such as pentanal, heptanal, 2-heptanal, decanal, in T7 Cool LED samples, suggests a lower resistance to the oxidative processes. On the contrary, the lower presence of PUFA in the Warm LED group was associated with a better resistance to oxidation as highlighted by a lower content of compounds belonging to the family of alcohols. Nevertheless, it would be necessary to carry out a sensory analysis to better define whether the observed changes in the volatile profile can cause variations in the aroma and flavor of the meat. The use of LED light is a good solution for poultry breeding due to the low environmental impact and energy consumption. However, the results of the present study suggest that different spectral ranges can have different effects on the chemical–nutritional characteristics of poultry meat.

## Figures and Tables

**Table 1:** Physical and chemical characterization of breast meat samples obtained from red chickens to different light treatments.

Chemical Physical Composition (%)	Control	Neutral LED	Cool LED	Warm LED
Cooking loss	$11.11^{\;a}\pm 1.43$	$12.79^{\;b}\pm 1.68$	$12.02^{\;a}\pm 1.38$	$11.36^{\;a}\pm 1.08$
Moisture	74.10±2.69	71.67±3.78	75.07±3.48	74.01±1.47
Dry matter	25.90±2.69	28.33±3.78	24.93±3.48	25.99±1.47
Total lipid ^**^	5.58±3.03	4.53±1.29	8.00±2.89	8.13±4.47

Data are reported as mean ± standard deviation (SD); ^a,b^ Different letters in the same row indicate significant differences (*p* < 0.05); ^**^ Data are reported on a dry-matter basis.

**Table 2:** Fatty acid profiles (%) of breast meat samples from red chickens exposed to different light treatments.

Fatty Acids	Control	Neutral LED	Cool LED	Warm LED
C14:0	0.64±0.50	0.42±0.13	0.47±0.08	0.45±0.08
C15:0	0.05±0.04	0.03±0.01	0.03±0.02	0.04±0.01
C16:0	22.96±2.09	22.27±0.76	22.26±1.80	22.86±1.98
C17:0	0.14±0.04	0.13±0.02	0.12±0.02	0.12±0.02
C18:0	$9.17\pm 1.74^{\;a}$	$9.10\pm 1.55^{\;a,b}$	$7.59\pm 1.26^{\;b}$	$9.14\pm 2.23^{\;a,b}$
C20:0	0.07±0.02	0.07±0.01	0.07±0.01	0.07±0.02
C22:0	$0.14\pm 0.07^{\;a,b}$	$0.16\pm 0.06^{\;a}$	$0.09\pm 0.04^{\;b}$	$0.13\pm 0.08^{\;a,b}$
C14:1	0.03±0.02	0.03±0.02	0.05±0.03	0.03±0.02
C16:1	$2.07\pm 0.86^{\;a}$	$2.15\pm 0.73^{\;a,b}$	$2.86\pm 1.03^{\;b}$	$2.18\pm 0.87^{\;a,b}$
C18:1, c9	$24.02\pm 3.01^{\;a}$	$24.79\pm 2.16^{\;a,b}$	$26.95\pm 2.73^{\;b}$	$24.41\pm 4.11^{\;a,b}$
C18:1, c11	1.82±0.33	1.84±0.31	1.59±0.43	1.88±0.37
C22:1	$0.11\pm 0.05^{\;a}$	$0.12\pm 0.03^{\;a}$	$0.07\pm 0.04^{\;b}$	$0.11\pm 0.05^{\;a,b}$
C18:2	29.30±2.52	29.18±1.62	30.31±2.25	28.97±1.67
C18:3	$2.29\pm 0.60^{\;a,b}$	$2.26\pm 0.45^{\;a,b}$	$2.72\pm 0.50^{\;a}$	$2.05\pm 0.65^{\;b}$
C20:4	$4.94\pm 2.14^{\;a}$	$5.20\pm 1.87^{\;a}$	$2.94\pm 0.69^{\;b}$	$5.12\pm 2.63^{\;a,b}$
Others	$2.24\pm 0.44^{\;a}$	$2.25\pm 0.27^{\;a}$	$1.89\pm 0.33^{\;b}$	$2.43\pm 0.64^{\;a}$
SFA	33.17±3.79	32.18±2.30	30.63±2.65	32.81±3.29
PUFA	$36.54\pm 2.35^{\;a}$	$36.64\pm 0.84^{\;a}$	$35.97\pm 2.48^{\;a}$	$31.02\pm 2.23^{\;b}$
MUFA	$28.05\pm 3.64^{\;a}$	$28.92\pm 2.52^{\;a,b}$	$31.52\pm 3.41^{\;b}$	$33.73\pm 2.49^{\;a,b}$

Data reported as mean ± standard deviation SD; ^a,b^ Different letters in the same row indicate significant differences (*p* < 0.05); SFA: saturated fatty acids; MUFA: monounsaturated fatty acids; PUFA: polyunsaturated fatty acids.

**Table 3:** Volatile profile of cooked breast meat samples obtained from red chickens exposed to different light treatments.

	T0				T7			
**VOC**	**Control**	**Neutral LED**	**Cool LED**	**Warm LED**	**Control**	**Neutral LED**	**Cool LED**	**Warm LED**
Aldehyde								
Pentanal	2.87±0.22	3.57±0.80	2.67±1.26	2.44±1.51	$1.20\pm 0.33^{\;a}$	$1.66\pm 0.30^{\;a,b}$	$1.80\pm 0.09^{\;b}$	$1.87\pm 0.44^{\;a,b}$
Hexanal	$44.65\pm 0.96^{\;a}$	$58.82\pm 7.80^{\;a,b}$	$64.54\pm 6.87^{\;b}$	$63.17\pm 9.51^{\;a,b}$	40.12±2.91	49.61±7.83	43.51±5.42	51.33±4.82
Heptanal	3.39±1.68	2.97±1.34	1.96±1.63	3.16±1.85	1.17±0.42a	$2.22\pm 0.54^{\;a,b}$	$2.67\pm 0.30^{\;b}$	$2.14\pm 0.55^{\;a,b}$
2-Heptanal	1.41±0.88	0.92±0.04	0.54±0.20	0.38±0.35	$0.29\pm 0.08^{\;a}$	$0.35\pm 0.16^{\;a,b}$	$0.46\pm 0.03^{\;b}$	$0.48\pm 0.26^{\;a,b}$
Octanal	$5.19\pm 0.32^{\;a}$	$3.70\pm 0.26^{\;b}$	$4.27\pm 1.50^{\;a,b}$	$4.01\pm 1.18^{\;a,b}$	4.45±1.41	4.81±1.22	5.15±0.24	5.13±0.61
2-Octenal	2.53±1.44	2.05±0.17	1.75±0.37	1.30±0.60	0.40±0.12	0.62±0.10	0.97±0.73	0.73±0.28
Nonanal	6.06±0.91	5.24±1.23	6.39±1.19	6.97±1.36	$9.53\pm 0.74^{\;a}$	$6.92\pm 0.91^{\;b}$	$8.43\pm 0.44^{\;a,b}$	$8.32\pm 0.98^{\;a,b}$
Decanal	2.63±1.76	0.51±0.13	0.62±0.19	0.44±0.11	$0.43\pm 0.14^{\;a,b}$	$0.44\pm 0.01^{\;a}$	$0.60\pm 0.04^{\;b}$	$0.81\pm 0.63^{\;a,b}$
Alcohol								
1-Pentanol	$2.01\pm 0.22^{\;a,b}$	$2.62\pm 0.33^{\;a}$	$1.26\pm 0.99^{\;a,b}$	$1.54\pm 0.47^{\;b}$	$2.47\pm 0.64^{\;a,b}$	$3.21\pm 0.80^{\;a,b}$	$4.01\pm 0.81^{\;a}$	$2.25\pm 0.49^{\;b}$
1-Heptanol	0.72±0.01	2.24±3.27	0.56±0.25	0.60±0.22	1.39±0.38	1.94±0.73	2.04±0.51	1.16±0.39
1-Octanol	n.d.	n.d.	n.d.	n.d.	$0.47\pm 0.13^{\;a,b}$	$0.96\pm 0.75^{\;a,b}$	$1.16\pm 0.36^{\;a}$	$0.24\pm 0.17^{\;b}$
1-Octen-3-ol	$21.30\pm 2.05^{\;a}$	$9.26\pm 5.14^{\;a,b}$	$9.01\pm 4.98^{\;a,b}$	$9.68\pm 4.58^{\;b}$	25.45±11.68	12.11±1.69	14.54±1.98	10.77±1.90
2-Octen-1-ol	0.43±0.38	0.33±0.14	0.21±0.21	0.09±0.11	$0.44\pm 0.12^{\;a,b}$	$1.26\pm 0.85^{\;a,b}$	$1.49\pm 0.53^{\;a}$	$0.41\pm 0.23^{\;b}$
1-Octyn-3-ol	$0.44\pm 0.30^{\;a,b}$	$0.38\pm 0.03^{\;a}$	$0.28\pm 0.06^{\;a,b}$	$0.25\pm 0.01^{\;b}$	0.14±0.05	0.14±0.05	0.16±0.07	0.23±0.05
Ketones								
2-Heptanone	0.04±0.01	0.02±0.01	0.03±0.03	0.03±0.03	$0.26\pm 0.07^{\;a}$	$0.59\pm 0.36^{\;a,b}$	$0.57\pm 0.03^{\;b}$	$0.17\pm 0.14^{\;a}$
2-Methyl 3-Octanone	$3.14\pm 0.26^{\;a}$	$4.73\pm 0.59^{\;b}$	$3.25\pm 0.91^{\;a,b}$	$2.63\pm 1.25^{\;a,b}$	5.55±3.73	6.83±1.57	6.25±0.88	5.06±2.02
Aromatic compounds								
Ethylbenzene	1.14±0.39	1.00±0.45	1.22±0.41	1.41±0.56	0.80±0.25	0.39±0.19	0.51±0.10	1.41±0.66
p-Xylene	1.45±0.67	1.40±0.31	1.18±0.34	1.27±0.21	1.09±0.31	1.43±0.57	1.72±0.24	1.56±0.07
Benzaldehyde	0.58±0.38	0.23±0.07	0.27±0.17	0.63±0.36	0.30±0.14	0.42±0.14	0.41±0.16	0.79±0.31
Ester								
Butanoic acid buthyl ester	n.d.	n.d.	n.d.	n.d.	1.61±0.52	1.68±0.60	1.57±0.52	2.14±0.99
Propionic acid buthyl ester	n.d.	n.d.	n.d.	n.d.	2.44±0.69	2.40±1.57	1.98±1.10	2.99±1.06

Data are reported as mean percentages of each volatile compound (VOC) ± standard deviation (SD); ^a,b^ Different letters in the same row indicate significant differences (*p* < 0.05); T0 = after cooking T7 = after 7 days of cooking; n.d. = not detected.

## Data Availability

The data presented in this study are available on request from the corresponding author.
